# Sensorimotor Connectivity in Parkinson’s Disease: The Role of Functional Neuroimaging

**DOI:** 10.3389/fneur.2014.00180

**Published:** 2014-09-24

**Authors:** Alessandro Tessitore, Alfonso Giordano, Rosa De Micco, Antonio Russo, Gioacchino Tedeschi

**Affiliations:** ^1^Department of Medical, Surgical, Neurological, Metabolic and Aging Sciences, Second University of Naples, Naples, Italy; ^2^MRI Research Center SUN-FISM, Second University of Naples, Naples, Italy; ^3^Institute for Diagnosis and Care “Hermitage Capodimonte”, Naples, Italy

**Keywords:** Parkinson’s disease, advanced MRI techniques, task-related functional MRI, resting-state functional MRI, seed approach, independent component analysis, sensorimotor network, dyskinetic motor complications

## Abstract

The diagnosis of Parkinson’s disease (PD) remains still clinical; nevertheless, in the last decades, the rapid evolution of advanced MRI techniques has made it possible to detect structural and, increasingly, functional brain changes in patients with PD. Indeed, functional MRI (fMRI) techniques have offered the opportunity to directly measure the brain’s activity and connectivity in patients with PD both in early and complicated stage of the disease. The aims of the following review are (1) to present an overview of recent fMRI reports investigating the activity and connectivity of sensorimotor areas in patients with PD using both task-related and “resting-state” fMRI analysis (2) to elucidate potential pathophysiological mechanisms underlying dyskinetic motor complications in the advanced stage of PD.

## Introduction

Parkinson’s disease (PD), the second most common neurodegenerative disease worldwide, is characterized by bradykinesia and at least one of tremor, rigidity, and postural instability (1). Although recent advances in neuroimaging have provided new insights into the pathophysiology of the disease, the diagnosis of PD remains still clinical, based upon the presence of cardinal motor symptoms (2). Neuroimaging of PD has been historically dominated by positron emission tomography (PET) and single-photon emission computed tomography (SPECT) studies using a variety of dopaminergic radiopharmaceuticals that focus on striatal measures of nigrostriatal neurons (3). Concurrently, fluorodeoxyglucose–PET has been used to image abnormal covariance patterns of cortical and subcortical regional metabolism that correlate with motor and cognitive impairment (4). On the other hand, conventional brain MRI is currently limited to the differential diagnosis between idiopathic PD and atypical or secondary parkinsonism or to prognosticate, and to locate the targets for functional neurosurgery (5). However, the use of 7-T MRI scanners has recently allowed to detect anatomical changes in nigral morphology in PD, which may represent, in the near future, a reliable MRI biomarker of PD diagnosis (6, 7). Nevertheless, in the last decades, the rapid evolution of advanced MRI techniques has allowed us to further investigate the progression of nigral and extra-nigral degeneration with greatly improved spatial resolution and a minimal invasiveness. Moreover, functional MRI (fMRI) techniques have offered the possibility to directly measure the brain’s activity and connectivity in patients with PD both in early and complicated stage of the disease. Therefore, morphological and fMRI techniques may shed lights on pathophysiological mechanisms of PD and its disease and treatment-related complications. The most commonly used method, among fMRI techniques, is the measurement of blood oxygen level dependent (BOLD) signal, based on the differences between magnetic characteristics of oxyhemoglobin (diamagnetic) and deoxyhemoglobin (paramagnetic). In the brain, neuronal activity increases consistently with blood flow and oxyhemoglobin and could be hence visualized by changes in the BOLD contrast. This neuroimaging technique enables to explore brain function and connectivity with high temporal and spatial resolution (8). More recently, fMRI in the absence of experimental tasks and behavioral responses, performed with the patient in a relaxed “*resting*” state (RS-fMRI), has allowed for the exploration of brain connectivity between functionally linked cortical regions (9), the so-called resting-state networks (RSNs). The most commonly reported RSNs are the default mode network, the fronto-parietal network, and the sensorimotor network (9), which is crucial for the execution of voluntary movements and functionally connects regions within the supplementary motor area (SMA) and the primary motor cortex (M1) (10, 11). The aims of the following review are (1) to present an overview of recent fMRI reports, which have investigated, in patients with PD, the activity and connectivity of the sensorimotor areas using both task-related and “resting-state” fMRI analysis, respectively, (2) to elucidate potential pathophysiological mechanisms underlying motor complications in the advanced stage of PD.

## Task-Related fMRI Studies

In the last decades, fMRI studies, employing motor tasks requiring motor selection and initiation (12–14), have consistently shown an abnormal activation of different areas of the motor network in patients with early and late-stage PD. The integrity of this network is required not only to perform a voluntary movement but also for readiness for a future motor task. These functional changes have been correlated both to bradykinesia and to the severity of the disease (i.e., Hoehn and Yahr stage). Specifically, these studies have commonly revealed hypoactivation of the SMA, and hyperactivation of cerebellum and other cortical motor regions, such as premotor (PMC), primary motor (M1), and parietal cortices in patients with PD compared to age-related healthy controls. It is noteworthy that these results have shown striking similarity with the pattern of altered cerebral metabolic activity observed with PET in patients with PD (15–18). Levodopa or apomorphine administration (12, 19), ventral posterolateral pallidotomy (20), or deep brain stimulation of the subthalamic nucleus (STN) (21) can relatively normalize the reduced activation of the SMA, and decrease the overactivation of other cortical regions. Because the SMA contributes to the preparation and execution of learned motor sequences (22–24), its decreased activation may be an important factor contributing to the lack of readiness and to the difficulty in initiating voluntary movements in patients with PD. Moreover, the hyperactivation of cortico-cerebellar regions may reflect a functional compensation for the defective basal ganglia in motor control. In other words, patients with PD may need compensatory activity of other motor circuits to overcome their difficulty in performing self-initiated movements (25). The compensation operated by the cerebellum could be achieved through the cerebello-thalamo-cortical loop or more directly through direct projections from the cerebellum to the basal ganglia (26, 27). Yu and colleagues (27) have found a significant negative correlation between the BOLD response in the putamen and the contralateral cerebellum, confirming cerebellar compensatory role in patients with PD. On the other hand, no significant correlation between the putamen and M1 was found and M1 hyperactivation was positively correlated only with the severity of upper limb rigidity. The increased connectivity between the M1 and pre-SMA has not only a compensatory role but could also reflect primary pathophysiological changes of PD, as a consequence of the inability to inhibit contextually inappropriate circuits (21, 28). Although several imaging studies on PD have reported similar findings (13, 19, 29), Buhmann and colleagues (19) have observed, in “drug-naïve” patients with PD while performing a simple, auditory-paced random finger-opposition task, a reduced activation of M1, which was partially restored only after levodopa intake. Thus, it is possible that the strengthened connectivity of the M1 and the related compensatory functional reorganization may be induced by the prolonged dopaminergic treatment. Functional reorganization of M1, indeed, is absent in “drug-naïve” patients with PD and hypoactivation of this brain region reflects the decreased input arising from the subcortical motor loop, which is partially restored by dopaminergic treatment.

## Resting-State fMRI Studies

The interpretation of these functional changes may be confounded by the fact that patients with PD have difficulty with performing motor tasks. RS-fMRI can overcome this problem by providing an index of connectivity across the whole brain. For this reason, a number of studies have applied RS-fMRI technique to investigate sensorimotor network connectivity in patients with PD (30–37). These reports have commonly demonstrated a disrupted functional integration in corticostriatal loops. Wu and colleagues (36), using a regional homogeneity approach, have demonstrated a decreased functional connectivity in the SMA, left dorsal lateral prefrontal cortex (DLPFC), and putamen and an increased cerebellar connectivity in patients with PD without dopaminergic medication for at least 12 h (“off-state”). The decreased SMA and basal ganglia connectivity was negatively correlated with the unified Parkinson’s disease rating scale (UPDRS) score, whereas a positive correlation was identified between increased cerebellar connectivity and the UPDRS score. Thus, this suggests that as the disorder progresses, resting-state neuronal activity in the SMA and basal ganglia becomes more abnormal and, at the mean time, the compensatory effect in the cerebellum is more significant. Previous fMRI and PET reports (19, 29, 38, 39) have already highlighted the involvement of DLPFC in patients with PD. The decreased connectivity of this region is probably related to the reduction in the attention to action and in performance monitoring typically observed in patients with PD. In another connectivity study by the same group (37), an increased connectivity in the right M1 and a decreased connectivity in the left putamen were confirmed in patients with PD. Moreover, an inferior parietal lobule (iPL) and a PMC disconnection with the pre-SMA were also detected. Thus, greater PD-related connectivity changes occur in networks linked to preparation and initiation rather than in those involved in motor execution. Indeed, iPL and PMC are related to the integration between motor selection and external information and selection of movements into a precise plan, respectively (22, 40, 41). The increased resting-state connectivity between the pre-SMA and right M1 confirms the potential compensation for the described decreased motor network connectivity in PD. In the first placebo-controlled RS-fMRI study, exploring the intrinsic sensorimotor network functional connectivity in “drug-naïve” patients with PD (42), we have demonstrated a decreased regional connectivity in the SMA in the “off-state,” which was partially restored only after levodopa administration. Moreover, a region of interest (ROI) analysis of the sensorimotor network functional connectivity in the basal ganglia and thalamus revealed that levodopa significantly increased the participation of these subcortical regions to the sensorimotor network activity. Finally, no statistically significant differences were detected between the groups in the M1 connectivity, confirming that the compensatory functional reorganization may be related to prolonged dopaminergic treatment rather than PD *per se*. Dopaminergic modulation of resting-state functional connectivity in “drug-naïve” patients with PD has also been evaluated using a correlation analysis with dopamine levels in the striatum, assessed quantitatively by FP-CIT PET (43). Choosing four ROIs, posterior cingulate cortex (PCC), putamen (anterior and posterior), and caudate, the authors found that the DLPC was the primary dopamine-dependent cortical region that was functionally connected with the anterior and posterior putamen. Moreover, patterns of dopamine-dependent positive functional connectivity varied depending on the location of the striatal seeds; dopamine-dependent functional connectivity from the caudate predominantly overlaid pericentral cortical areas, whereas dopamine-dependent structures that were functionally connected to the posterior putamen predominantly involved cerebellar areas. Finally, there were cortical areas where the cortico-cortical or striato-cortical connectivity were negatively associated with the dopaminergic status in the posterior putamen. Therefore, dopamine deficiency may lead to alterations in resting-state functional connectivity and reorganization of striato-cortical functional network in patients with PD. This may be one of mechanisms underlying impaired sensorimotor integration in PD. According to Braak staging (44), the pathologic process of PD, occurring primarily in the brainstem, pursues an ascending course reaching the neocortex in the final stage; thus, subcortical involvement prevails throughout the course of PD and functional changes in the basal ganglia lie at the heart of PD. Based on this a number of seed based RS-fMRI studies (31, 32, 45), using left and right putamen, caudate, and amygdala as seeds, have shown a significantly reduced connectivity within mesolimbic–striatal and corticostriatal loops in “drug-naïve” PD patients. In these studies, both anterior and posterior putamen showed a decreased connectivity pattern with their contralateral putamen and mesolimbic regions, especially in amygdala, hippocampus, olfactory area, and posterior rectus, whereas the posterior putamen presented a more prominent decreased pattern extending to sensorimotor cortex. The caudate connectivity pattern was relatively spared. Functional connectivity analysis of the amygdala also showed coherent reduced connectivity pattern with the putamen. Moreover, putamen connectivity with amygdala, a limbic region crucial for emotional processing (45) was significantly correlated with non-motor symptom scale (NMSS) total score and NMSS mood subscale score. No compensatory increased functional connectivity was found in this study. Baudrexel and colleagues (30) set out to define the differences of resting-state STN functional connectivity networks between patients suffering from early stage PD and healthy controls using this straightforward seed-region approach. STN was selected as seed region because it is both part of the slow “indirect” and a fast “hyper-direct” functional cortico-subcortical loop, and it is currently the most effective target for DBS in patients suffering from advanced PD (46). The analysis revealed an increased functional connectivity between right and left STN and bilateral M1, PMC, SMA, and also primary sensory regions in patients with PD, confirming the well-established results from electrophysiological recordings, which have demonstrated excessive synchronization in basal ganglia-cortical circuitries at a vastly different temporal scale (47–49). This fMRI resting-state study provides an additional evidence that a pathologic subthalamic–cortical coupling might be a crucial factor in the pathophysiology of PD (50). These results are in line with the original model of BG functioning proposed by Alexander and DeLong (51, 52); dopamine depletion, indeed, causes suppression of the “direct” cortical-BG feedback loop (cortex–striatum–internal globus pallidum/SNr–thalamus–cortex) and a release of the “indirect” loop (cortex–striatum–external globus pallidum–STN–GPi/SNr–thalamus–cortex) both, resulting in hyperactivity of the STN. Although RS-fMRI studies have mainly provided insights into the pathophysiological mechanisms underlying motor and non-motor symptoms of PD, a recent study (53) has demonstrated that changes in basal ganglia network (BGN) connectivity could help us to differentiate patients with PD from healthy controls. Using a BGN template derived from 80 elderly controls, patients with PD showed a reduced functional connectivity in a wide range of BGN areas (such as putamen, caudate, anterior thalamus, DLPC, and precuneus). This functional alteration was clearly improved by levodopa administration. Moreover, average BGN connectivity was able to differentiate patients with PD from controls with 100% sensitivity and 89.5% specificity, confirming the potential role of RS-fMRI connectivity as a biomarker in early PD.

## Sensorimotor Connectivity in Dyskinetic Phase

Long-term levodopa treatment is complicated by the gradual development of involuntary movements referred to as levodopa-induced dyskinesias (LID). Recent studies have evidenced a substantial progress in understanding the cellular and molecular mechanisms, which underlie dyskinesias (54, 55). Hypersensitivity of striatal medium spiny neurons to pulsatile dopamine receptor stimulation during task-related corticostriatal activation of glutamate receptors seem to plays a crucial role to the development of these motor complications (56). Neuroimaging studies of dyskinesias in humans are sparse, because dyskinesias cause movement artifacts impairing data quality. Cerasa and colleagues (57), comparing patients with PD with and without LID during execution of externally and internally triggered visuomotor tasks, showed significant SMA hyperactivity and hypoactivity in the right inferior prefrontal gyrus only in patients with LID. However, it remains unclear how intake of levodopa modulates neural activity in patients with dyskinesias. More recently, Herz and colleagues (58) performed a task-related fMRI experiment in the time window between the intake of levodopa and the onset of motor complication in dyskinetic vs. non-dyskinetic patients with PD, which were asked to produce a mouse click with the right or left hand or no action (No-Go). During No-Go trials, patients with PD, who would later develop dyskinesias, showed an abnormal gradual increase of activity in the pre-SMA and the bilateral putamen during the first 20 min after levodopa intake. This rapidly emerging hypersensitivity of putamen and pre-SMA in the context of movement suppression (No-Go) and in the pre-dyskinesia period might reflect an unphysiological facilitation or impaired inhibition, via striatal D2-type receptors (56), of motor programs, resulting in aberrant activity in interconnected cortical areas.

## Conclusion

In conclusion, although the diagnosis of PD remains still clinical, functional imaging studies can provide great insights into connectivity changes and pathogenic processes in PD. Task-related fMRI studies have shown an abnormal activation of different areas of the motor network in patients with early and late-stage PD related to cardinal clinical features. More recently, RS-fMRI techniques have been used to investigate sensorimotor network connectivity in patients with PD confirming a disrupted functional integration in corticostriatal loops. Therefore, in the near future, the practical application of these techniques may provide a better understanding of disease- and treatment-related complications and a reliable MRI biomarker for an early diagnosis of PD.

## Conflict of Interest Statement

The authors declare that the research was conducted in the absence of any commercial or financial relationships that could be construed as a potential conflict of interest.
